# Human Activity Recognition via Hybrid Deep Learning Based Model

**DOI:** 10.3390/s22010323

**Published:** 2022-01-01

**Authors:** Imran Ullah Khan, Sitara Afzal, Jong Weon Lee

**Affiliations:** Mixed Reality and Interaction Lab, Department of Software, Sejong University, Seoul 05006, Korea; imrankhan@sju.ac.kr (I.U.K.); sitara.afzal@yahoo.com (S.A.)

**Keywords:** human activity recognition, convolutional neural network, deep learning, long short-term memory, machine learning, skeleton data

## Abstract

In recent years, Human Activity Recognition (HAR) has become one of the most important research topics in the domains of health and human-machine interaction. Many Artificial intelligence-based models are developed for activity recognition; however, these algorithms fail to extract spatial and temporal features due to which they show poor performance on real-world long-term HAR. Furthermore, in literature, a limited number of datasets are publicly available for physical activities recognition that contains less number of activities. Considering these limitations, we develop a hybrid model by incorporating Convolutional Neural Network (CNN) and Long Short-Term Memory (LSTM) for activity recognition where CNN is used for spatial features extraction and LSTM network is utilized for learning temporal information. Additionally, a new challenging dataset is generated that is collected from 20 participants using the Kinect V2 sensor and contains 12 different classes of human physical activities. An extensive ablation study is performed over different traditional machine learning and deep learning models to obtain the optimum solution for HAR. The accuracy of 90.89% is achieved via the CNN-LSTM technique, which shows that the proposed model is suitable for HAR applications.

## 1. Introduction

HAR gained more attention from researchers in video analysis and its different applications in various domains such as indoor gym physical activities [1], surveillance systems [2], and health care systems [3]. In the light of literature, activity recognition is performed based on wearable sensors and vision sensors. In wearable sensors based HAR, many sensors are attached to a subject’s body for a prolonged period, which is cumbersome for the subject’s body and the subject can’t move comfortably because of many wire connections, as well as it is expensive in terms of energy consumption and device configuration. Instead of focusing on wearable sensor based HAR, numerous studies incorporated video sensor technologies like RGB cameras to monitor and recognize human activity. The current literature studies focus to recognize activities using video sequences collected by standard RGB cameras and surveillance cameras [4,5]. Recognition of activity through common cameras may be a problem of difficulty in recognition due to low light environment or darkness. To avoid the problem of light variation, a low-cost RGB-D camera, such as Microsoft Kinect, has been made possible the recent advancement in activity recognition. Kinect-based action recognition tackles the light-environment problem and accurately tracks the skeleton joints during activity, and it also offers a variety of information, such as depth and skeleton information, that a standard video camera failed to provide. In addition, RGBD data from the Kinect sensor may be utilized to create a human skeleton model with body joints. However, human actions are the collection of various joints that move over time and these joint data can be used for the recognition of activity.

Numerous studies demonstrated the positive influence of physical activities on people’s quality of life, especially for elderly people. The involvement of the elder people in particular physical activities has positive effects on mental state, satisfaction, quality of life, and physical well-being [6]. Due to the current situation of COVID-19, the government of many countries-imposed lockdowns and home confinement which constrained the people to stay at home and avoid physical activities in public places. The outbreak of Coronavirus has begun in December 2019, and it spread out by human-to-human interaction which results in huge loss of human life. According to the recent report of the World Health Organization (WHO), there are almost 270 million positive cases and 5.3 million deaths occurs till now due to COVID-19 disease [7]. To prevent the spread of Coronavirus infection, many safeties measure has been taken worldwide such as home confinement, banning gatherings and visiting crowded public places, and avoiding outdoor activities. Many countries enforced lockdown in the country to control the spread of COVID disease which limits the participation of people in healthy activities. People are recommended to stay at home and avoid going outside for exercises or other physical activities in these situations. Therefore, in this paper, we developed an indoor monitoring system for physical activity recognition.

HAR is not a novel concept and several studies have been conducted in this domain. However, the current literature is mainly focusing on traditional machine learning algorithms which required handcrafted features engineering with lower accuracy. Furthermore, some authors proposed deep learning based HAR systems by directly migrating these methods from other domains to the HAR domain without in-depth analysis. The current deep learning-based approaches are mainly focusing on CNN and RNN variant architectures. CNN-based architectures are designed for spatial information extraction while RNN-based architectures are specially designed for temporal features extraction. The HAR data is time-series data including spatial and temporal information which requires a robust model with the potential to extract both information at a time. Therefore, in this work, we developed a hybrid model combining CNN with LSTM with the potential to extract both features at a time and to recognize several physical activities. Furthermore, we also contribute a new dataset collected from many participants who perform 12 types of different physical activities which helps in maintaining the strength, balance, and flexibility of the human body. The techniques used in the research mainly use the skeleton joints data which is extracted through the Kinect V2. The process is mainly divided into many steps. The initial step is the collection and pre-processing of 2D joint data through the Kinect which are fed forwarded to 1D CNN layers for spatial features extraction. The output of CNN is then inputted to LSTM network LSTM for temporal features learning followed by the fully connected layer for final recognition. The main contributions of the proposed work are given below:

1. We proposed an indoor activity recognition system to efficiently recognize different types of activities to improve the physical and mental health of an individual.
2. We developed a hybrid approach for the recognition of physical activity which integrates CNN and LSTM, where CNN layers are utilized to extract spatial features followed by the LSTM network for learning temporal information.
3. We performed a detailed comparative analysis of various machine learning and deep learning models to select the best optimal modal for activity recognition.
4. No publicly available dataset provides home base physical activities; therefore, we contribute a new dataset comprising 12 different physical activities performed by 20 participants.

The remainder paper is arranged as; literature study of HARis discussed in Section 2, proposed methodology, and dataset description are explained in Section 3. Experimental results and evaluations of the proposed model are described in Section 4, and Section 5 concluded the paper.

## 2. Literature Review

HAR is not a novel concept and numerous studies have been conducted in this area, however, in this section, we are focusing on the recent literature developed for HAR. The current literature of HAR is based on machine learning and deep learning. In machine learning Sumaira et al. [8] performed a comparative analysis of several models for HAR using 2D-skeletal data. The authors used the OpenPose library to extract appearance and motion characteristics from 2D landmarks of human skeletal joints and compared the result of five supervised machine learning approaches such as support vector machine (SVM), Naive Bayes (NB), linear discriminant (LD), K-nearest neighbors (KNNs) and feed-forward backpropagation neural network to recognize four different activity classes such as sit, stand, walk, and fall, while the best performance was achieved through KNNs technique. Guangming et al. [9] conducted research based on an online Continuous Human Action Recognition (CHAR) algorithm which relies on skeletal data extracted through Kinect depth sensor. An online classification technique using a variable-length maximum entropy Markov model (MEMM) based on likelihood probabilities is utilized for continuous activity recognition. In contrast to previously reported CHAR approaches, the suggested algorithm does not require prior detection of the start and finish points of each human activity. According to experimental findings on the MSR Daily Activity 3D dataset and Cornell CAD-60 dataset, their proposed method is very efficient for continuous human activities recognition. Another technique [10] uses skeletal data from a depth camera and developed a machine-learning algorithm to recognize the human activity. In comparison to previous techniques, each activity is represented using a distinct number of clusters that are retrieved independently from activity instances. These models are created using a multiclass SVM that has been trained on two publicly available datasets, the CAD-60 and the TST using the SOM optimization. These numbers can change depending on the input sequence and activity, resulting in clusters that are dynamically generated. Youssef et al. [11] developed a skeleton-based technique to characterize the spatial-temporal features of a human activity sequence utilizing Minkowski and cosine distances between joint data extracted through Microsoft Kinect. The model is trained and evaluated on two publicly available datasets such as MSR Daily Activity 3D and Microsoft MSR 3D Action datasets using the Extremely Randomized Tree technique. The results are highly encouraging, indicating that utilizing open-source libraries and a low-cost depth sensor, the trained model was utilized to construct a monitoring system for the elderly. 

Another group of researchers [12] proposed a pose descriptor for differential quantities encoders as well as for taking the information of human joint’s posture in a frame sequence efficiently. They utilized the k-nearest neighbor method to join the descriptor, but their results are non-parametric and low-latency recognition. In [13], the authors presented the sequence of most informative joints features, and represent the information of skeletal joints for each action. They choose the joints based on the mean and variance of the angular-joint trajectories for a given action sequence. The authors of [14] presented the Eigen Joints features which comprise 3D position contrasts in joints to describe activity data. The components were designed as a blend of three-element channels: the posture-feature channel and the movement include a channel for encoding the spatial part of the grouping and the offset feature for addressing the posture contrast amongst frames. The principal component analysis (PCA) was applied to these three channels to figure the Eigen Joints features. They utilized the Naïve Bayes classifier for activity recognition. In [15], the authors combined 3D joint position differences inside a casing with the joint differences from the initial frame of an action to produce outline features. The features of these frames are concatenated to make a frames sequence. In [16], every appendage of the human skeleton is encoded into a state through a Markov random field by considering the spatial information and the fleeting setting data from the past outline. The encoded elements of individual appendages are then averaged for representing the skeleton information. A covariance grid for skeletal joint areas over the long haul has been utilized in [17] as a discriminative descriptor for a sequence. Various covariance frameworks over aftereffects were sent to encode the connection amongst joint development and time. L. Arthi et al. [18] proposed a sample of fusion network (SFN). They employed an adaptive weighting approach to enhance the complementation amongst samples and new samples generated by utilizing a sample fusion network. SFN enhances the performance of the HAR network while training the network. For their findings, they attained 90.75% accuracy on the NTU data samples by utilizing cross-view protocol. However, these algorithms are based on machine learning which required hand-crafted features extraction with limited generalization abilities which causes parameters non-convergence and network instability. Hence, these challenges encourage the researchers and domain experts to reconsider HAR based on deep learning.

Deep learning based HAR is already developed in the recent literature. For instance, Julieta et al. [19] focus on human motion by utilizing the recurrent neural network, the goal is time-dependent representations to perform tasks including short-term prediction as well as long-term human motion synthesis. For their finding, they also utilize other state-of-the-art approaches to compare the results of these approaches with the enhanced recurrent neural network approach. Chao li et al. [20] proposed a framework that is an end-to-end CNN features learning framework. They utilized a hierarchical approach to learned co-occurrence features having distinct contextual information. Initially, they encode point-level information independently and then present the semantic representation in spatial as well as temporal. In their findings, they proposed a global-spatial approach that can learn superior joint information. Maosen Li et al. [21] proposed two graphs scale to capture the relationships amongst body joints and parts. They presented a symbiotic neural network with a backbone, action recognition head, and motion prediction head. These two heads are connected and improve the joint recognitions. To extract the temporal as well as spatial features, they utilize multiscale CNN. The joint scale graphs and structural graphs capture the actions and physical constraints respectively. Comparatively the performance of the deep learning-based model is better than machine learning-based algorithms however HAR data is time-series data that includes spatial and temporal information which required a robust model with the ability to learn both information of human activity. Therefore, in this work, we developed a hybrid model for HAR with the potential of spatiotemporal feature extraction for effective HAR.

## 3. Proposed Method

In this work, we conduct a detailed ablation study, developed a new dataset, and a novel deep learning-based hybrid model to monitor and recognize human physical activity in an indoor environment. This section briefly describes the internal architecture of the proposed work, proposed dataset, and comparative study.

### 3.1. Dataset Collection & Preparation

**Figure 1:**
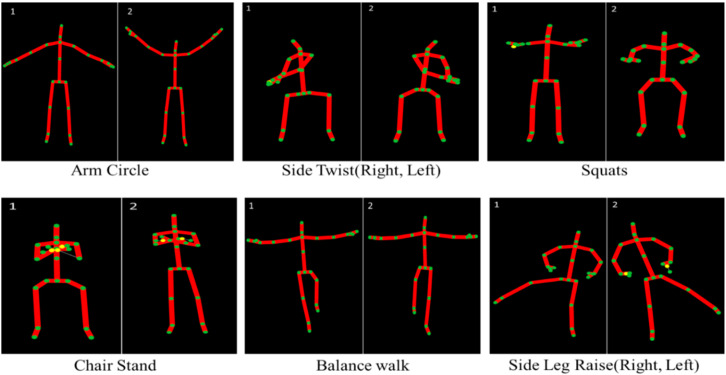
The extracted skeleton of the human body while performing different activities.

This section provides a detailed analysis of the collection and refinement of data. In this paper, we have generated our dataset. The proposed dataset includes 12 different activities taken from 20 individuals aged between 25–35 years. For the collection of this data, we used Microsoft’s motion Kinect sensor V2 which can extract 25 different joints from the human body as shown in Figure 1. 

**Figure 2:**
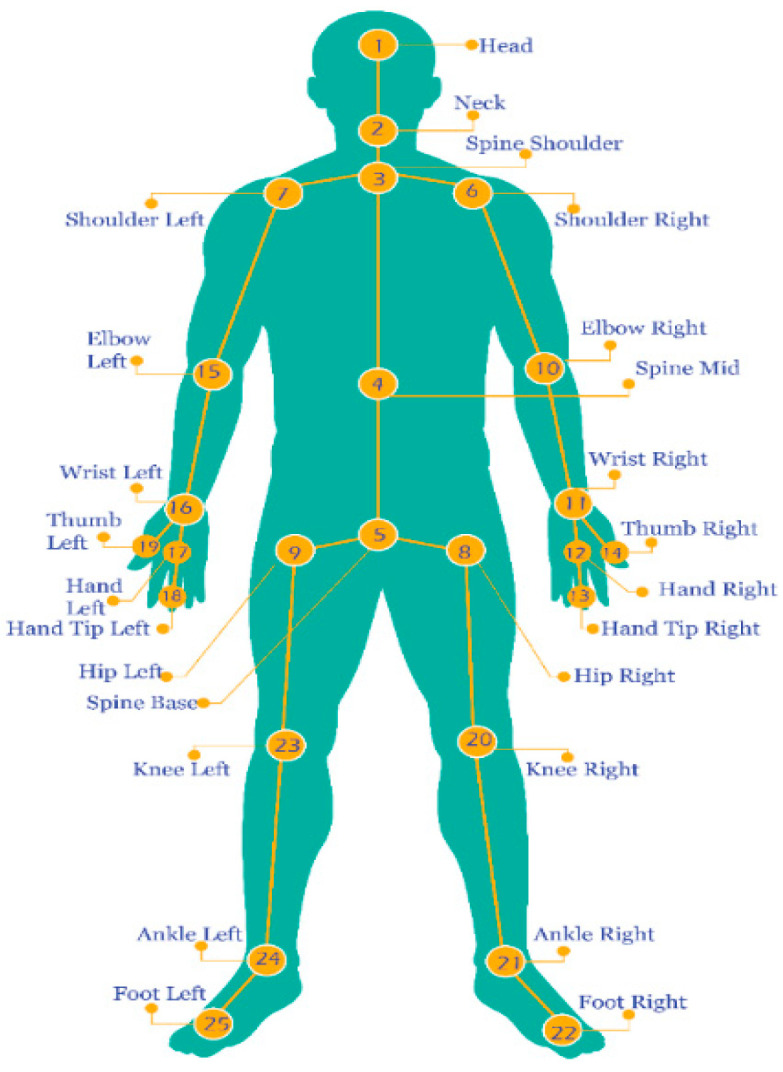
Different skeleton joints of the human body are extracted through sensors.

We extract the *x*-axis and *y*-axis values from all the joints of the human body and save them in CSV files. We collected a dataset from 20 different participants and every participant perform an activity for 10 s. There are 200 samples of each activity where every participant performs each activity for 10 times (120 samples per participant). The human skeleton joints are extracted and stored in the following order shown in Figure 2. Each activities files are combined and labeled with their class as shown in Table 1. After labeling all activities data, all these files are further combined in a single training file. Table 1 shows the detailed description of the individuals and activities during data collection.

### 3.2. Skeleton Joints Position

Human skeleton joints are extracted using Kinect V2 sensor. We extracted the human joints by using Discrete Gestures Basics WPF SDK. We capture the joint data through the Kinect Body View script and save it in CSV files. The Kinect V2 can detects 25 joints of the body and it is stored in following order such as Head, Neck, Spine Shoulder, Spine Mid, Spine Base, Shoulder Right, Shoulder Left, Hip Right, Hip Left, Elbow Right, Wrist Right, Hand Right, Hand Tip Right, Thumb Right, Elbow Left, Wrist Left, Hand Left, Hand Tip Left, Thumb Left, Knee Right, Ankle Right, Foot Right, Knee Left, Ankle Left, and Foot Left. The joints are labeled as 1, 2, 3, 4…, 25 as given in Figure 2.

### 3.3. Machine Learning Techniques

**Figure 3:**
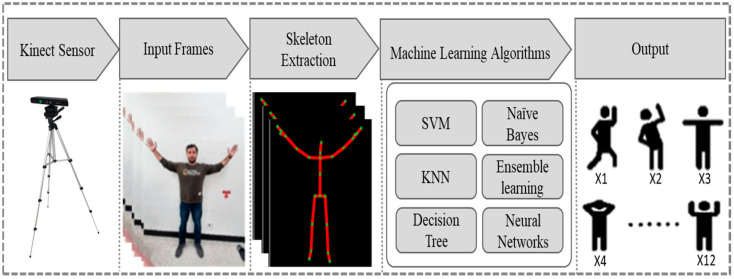
Activity recognition through different Machine learning algorithms.

We use many traditional machine learning classifiers for the experimental evaluation of our dataset. The data is divided into five different types of sequences such as 30 frames (1 s), 60 frames (2 s), 90 frames (3 s), 120 frames (4 s), and 150 frames (5 s) frames sequence. To capture the unique features of action or activity, complex machine learning-based models such as various flavors of SVM [22] (Linear SVM (LSVM), Quadratic SVM (QSVM), Cubic SVM (CSVM), Fine Gaussian SVM (FGSVM), Medium Gaussian SVM (MGSVM), Coarse Gaussian SVM (CGSVM)), KNN [23] (Fine KNN (FKNN), Medium KNN (MKNN), Coarse KNN (CRSKNN), Cosine KNN (CSNKNN), Cubic KNN (CBCKNN), Weighted KNN (WKNN)), Decision Tree [24] (Fine Tree (FT), Medium Tree (MT), Coarse Tree (CT)), Linear Discriminant (LD) [25], Naïve Bayes [26] (Gaussian Naïve Bayes (GNB), Kernel Naïve Bayes (KNB)), Ensemble classifiers [27] (Ensemble Boosted Trees (EBST), Ensemble Bagged Trees (EBGT), Ensemble Subspace Discriminant (ESD), Ensemble Subspace KNN (ESKNN), RUSBoosted Trees (ERUSBT)), and Neural Networks (NN) [28] (Narrow Neural Network(NNN), Medium Neural Network(MNN), Wide Neural Network(WNN), Bilayered Neural Network (BNN), Trilayered Neural Network(TNN)). The performance of these modes is evaluated on the proposed dataset to choose the best optimal model for HAR. The overall workflow of machine learning classifiers is shown in Figure 3.

SVM is a supervised learning model that strives for maximal margin separation with a little amount of training data. The training set is used to generate a plane and hyperplane for both the linear classification and for nonlinear classification respectively, that distinguishes data from various classes. The plane or hyperplane can clearly classify the data into their actual classes. The KNNs method is a supervised learning technique that classifies the outcome of a new sample query based on the majority of K-Nearest Neighbor categories. It is one of the most widely used pattern recognition algorithms and its goal is to categorize a new item based on its characteristics and training data. The neighborhood classification was utilized as the prediction value of the new query sample using the K-Nearest Neighbor method (classification approach that uses the feature space’s closest training samples). A Decision Tree is a supervised machine learning technique that can be utilized for both regression and classification problems and the main objective of decision trees is to construct a training model which is used to identify the testing variable’s class or value by learning basic decision trees gained from training data. The samples are categorized using decision trees by organizing them along the tree from the root to the leaf node, which classifies the samples. Each node in the tree represents a test case for a certain feature, and every descending edge from the node represents the test case’s possible prediction. This is a cyclical process that happens for each subtree rooted at the new node.

Another type of machine learning classifier is Linear Discriminant, which is developed based on finding a linear combination of variables (predictors) that best differentiates two target classes. In this algorithm, the mean vector, covariance matrices, and probability of classes are calculated in the initial step while pooled covariance matrices and linear model coefficients are calculated in the second step thatcomputes the Mahalanobis distance. This distance shows the overlapping between classes which means the variation between classes via linear model. The Naïve Bayes algorithm is used for prediction where each class is independent of one another, however, it performs well in real even when this statement is imprecise. It divides data into two categories, first is the training step in which it calculates the parameters of a probability distribution using the training data, assuming that predictors are mutually independent of the class. In the second step, it calculates the posterior probability of a sample related to each class for any unknown test data. The test data is then classified using the highest posterior probability. Moreover, we use ensembles classifiers that integrate several models and improve the robustness and generalization ability of a classifier. In comparison to a single model, this method provides a higher predictive performance. The technique used by ensemble classifiers is mainly comprised of the majority voting method and finding the average of different predictors outputs.

The Neural Network simulates a large number of interconnected processing units that look like complex structures of neurons. The processing units are arranged layers wise such as an input layer with units representing the input fields, single or multi hidden layers, and an output layer with a unit or units representing the final output. The components are connected using a variety of weighted connections (or weights). The first layer receives input data, and values are transmitted from each neuron to the neurons in other layers and the last output layer will eventually give a result.

Artificial Neural Network (ANN) is an advanced type of machine learning inspired by the human nervous system. Multilayer perceptron (MLP) is a type of neural network which consists of input layers, hidden layers, and output layers. Every neuron of each layer is connected to each neuron in the previous layer and next layer. The value obtained from the earlier layers is added with weights for every neuron individually and an extra bias term is added. These values are summed up and multiplied with the activation function for the final output. Different types of activation functions are used in ANN such as “sigmoid”, “softmax”, Rectified Linear Unit “ReLU” and “Tanh”. Various types of optimizers can be used in ANN adaptive moment estimation (Adam), “Adagrad” and RmsProp, etc., in our case we use Adam optimizer. 

### 3.4. Convolutional Neural Network (CNN)

Over the last two decades, CNNs have been actively used and achieved astonishing performance for various computer vision-related real-world problems that include activity recognition [29], object detection [30], speech recognition [31,32], and image enhancement [33]. The key factor behind the betterment of CNNs for computer vision problems is their architectural design including convolutional, pooling, normalization, and fully connected layers that extract progressive yet semantically rich features from the input data [34]. Generally, a convolutional layer processes the input image and produces a batch of 2D feature maps containing spatial features, where the pooling layer simply scales down the extracted feature maps by applying down-sampling operations (i.e., max pooling, min pooling, or average pooling operations). Where the mathematical representation of a convolutional layer is given below.

(1) $$C_{i,j,k}^{l}=f(\left(w_{k}^{l}{)}^{T}\;x_{i,j}^{l}\;\right)+b_{k}^{l}\;$$

where $b_{k}^{l}$ is a bias term of a kth CNN filter in the 1st layer, $x_{i,j}^{l}$ represents the input region in the 1st layer. The normalization layer is usually used before the activation function that normalizes the input values and leads to more accurate activation. The fully connected layer parses the extracted feature maps from 2D to 1D feature vectors, which are then forwarded to the classification layer or output layer (i.e., softmax) and results from the computed list probabilities. Inspired by the work presented in [35,36], we propose a One-Dimensional (1D) CNN architecture for the problem under the observation test with different settings for efficient classification of predefined indoor activities.

### 3.5. Long-Short Term Memory (LSTM)

**Figure 4:**
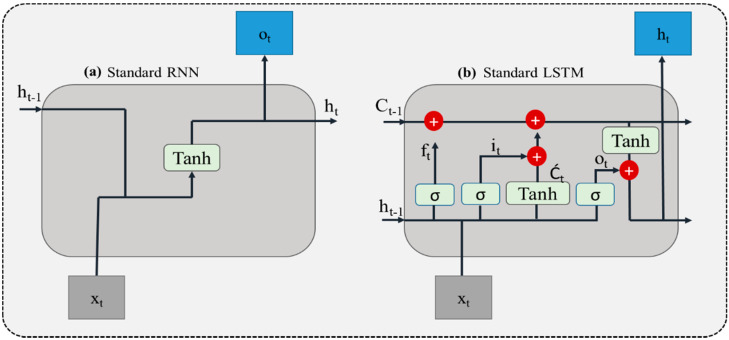
(**a**) Represents the standard RNN unit, (**b**) represents the standard LSTM unit.

Despite the robustness and efficiency, CNN-based approaches can only be used for fixed and short sequence classification problems and are not recommended to use for long and complex time series data problems. Mostly a problem having sequential analysis over time such as anomaly recognition [37,38] speech recognition [39,40], person re-identification [41], Energy forecasting [42,43,44,45], machine translation [46], and activity recognition from sensor data [47] used a special kind of neural network called Recurrent Neural Network (RNN) specifically designed for sequential data analysis having the ability to extract the hidden pattern from sequential data. Generally, the RNN network analyzes the input hidden sequential pattern by concatenating the previous information with current information from both spatial and temporal dimensions and predicting the future sequence [48]. Although RNN can extract the hidden time-series patterns in sequential data (i.e., sensor, audio, or video data), it is unable to remember/hold long information for a long time and usually fails to deal with the problems having long-term sequences [49,50]. Such a type of problem is referred to as gradient exploding or vanishing gradients, which can be overcome with a special kind of RNN named Long Short-Term Memory (LSTM) having the capability to remember the information for a long period [51]. The internal architecture of LSTM includes several gates (including input, forget, and output gate), where each gate processes the input from the previous gate and forward it to the next gate thereby controlling the flow of information towards the final output [52] Figure 4 demonstrates the standard unit of the (a) RNN and (b) LSTM. All gates are usually controlled by a sigmoid or *tanh* activation function, for instance, the input gate $i_{t}$ is responsible to update the information. The forget gate process the input information from the input gate $i_{t}$ and the state of previous cell $C_{t-1}$, it also removes the information from the current state $C_{t}$ when needed. Whereas the output gate $o_{t}$ forwards the final output to the next LSTM unit and holds the output value for the next sequence prediction. On the other hand, recurrent unit ${Ċ}_{t}$ estimates the state of pervious cell $C_{t-1}$ and current input value $x_{t}$ using *tanh* activation function. Whereas the value of $h_{t}$ can be computed by the scalar product of $o_{t}$ and *tanh* of $C_{t}$. Finally, the ultimate output can be obtained by passing $h_{t}$ to the softmax classifier. Mathematically, the operations of the above-mentioned gates can be expressed as follows:

(2) $${ƒ}_{t}=\Phi \;\left({Ŵ}_{f}\;\cdot \;\left[h_{t-1},\;x_{t}\right]+B_{f}\right)$$

(3) $$i_{t}=\Phi \;\left({Ŵ}_{i}\;\cdot \;\left[h_{t-1},\;x_{t}\right]+B_{i}\right)$$

(4) $${Ċ}_{t}=tanh\;\left({Ŵ}_{C}\;\cdot \;\left[h_{t-1},\;x_{t}\right]+B_{C}\right)$$

(5) $$C_{t}=f_{t}\;x\;C_{t-1}+i_{t}\;x\;{Ċ}_{t}$$

(6) $$o_{t}=\Phi \left({Ŵ}_{o}\;\cdot \;\left[h_{t-1},\;x_{t}\right]+B_{o}\right)$$

(7) $$h_{t}=o_{t}\;xtanh(\Phi \left(C_{t}\right)$$

(8) $$Output=softmax\left(h_{t}\right)$$

### 3.6. Proposed CNN-LSTM Model

**Figure 5:**
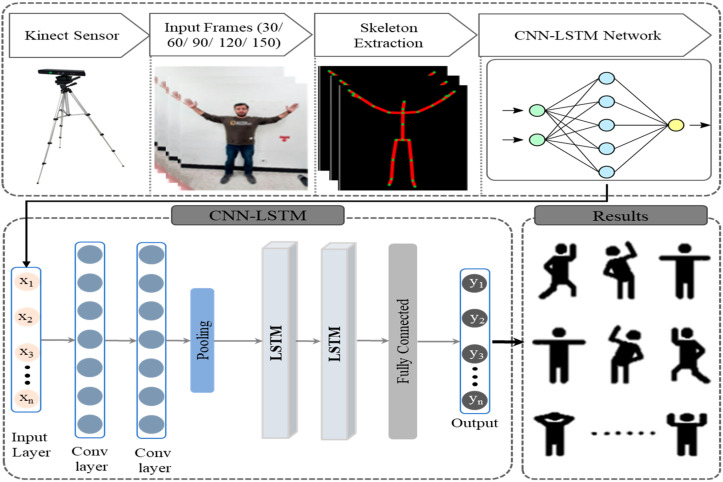
The overall framework of the proposed hybrid CNN-LSTM approach.

We propose the hybrid approach in which features are extracted from the layers of the first model and then forward to another model for learning and modeling. As 1D CNN acquired consideration of researchers due to its performance by extracting the spatial and discriminative feature from data. However, LSTM has been used by many researchers which shows its efficiency in sequential and time-series data. By combing these two models, we extract features through 1D CNN and then forwarded these features to LSTM for learning and modeling. The first two layers of 1D CNN has different filter size such as in the first layer, the filter size is 64, while in the second layer the filter size is 128. Other than filter size, the kernel size of both layers is 3 and the activation function used in both layers is the ReLU activation function. These two layers are followed by the Max pooling layer with a pool size of 2. These features form the CNN layers are passed through two LSTM layers with the same cell size of 64 in each layer. The LSTM layer is followed by the flatten layer and dense layer with a softmax activation function. The optimizer used in this approach is Adam with a learning rate of 0.0001. Themain framework of the proposed model is shown in Figure 5. The parameter setting of the proposed model is given in Table 2.

## 4. Experimental Results

In this section, we perform several experiments to evaluate the performance of a machine learning classifiers and deep learning models on a different sequence of data. All the machine learning classifiers are analyzed in MATLAB 2021a, while deep learning experiments are performed in python, using Keras framework with backend TensorFlow and Scikit-learn in this research implementation. Five different types of experiments are performed on the various frames sequence of data such as 30 frames sequence, 60 frames sequence, 120 frames sequence, and 150 frames sequence using both machine learning classifiers and deep learning models.

### 4.1. Dataset Descriptions

To evaluate the performance of our technique, we create our dataset which consists of 12 activities collected from 20 different participants. Every participant is directed to perform 12 different physical activities which include different exercises related to strength exercises, balance exercises, and flexibility exercises that can be also helpful in maintaining the mental health of an individual. More specifically these physical activities include *Overhead Arm Raise*, *Front Arm Raise*, *Arm Curl*, *Chair Stand*, *Balance Walk*, *Side Leg Raise (Right*, *Left)*, *Shoulder*, *Chest*, *Leg Raise (Forward*, *Backward)*, *Arm Circle*, *Side Twist (Right*, *Left)*, *Squats.* Every individual performs an activity for 10 s with a 30-frame rate and the Kinect V2 extracts the joint data from the human skeleton and saves it in CSV files. After completing the data collection, the data is arranged in such a format where all the individual’s data of the same activity is appended in one file. Moreover, the data is divided into different sequences such as 1 s (30 frames), 2 s (60 frames), 3 s (60 frames), 4 s (120 frames), and 5 s (150 frames), and all the activities data are then labeled according to their classes.

### 4.2. Evaluation Metrics

In this work, we used three types of evaluation matrics such as Accuracy, Precision, and Recall to evaluate the performance of each model. Activity can be classified as True Positive (*TP*) and True Negative (*TN*) in case of correctly recognized while in case of incorrect classification, it can be False Positive (*FP*) or False Negative (*FN*). Other performance matrices are derived from *TP* or *TN*. Given $TP=\sum_{k=1}^{n}TP_{k}$ represents the addition of all true positive samples,$\;TN=\sum_{k=1}^{n}TN_{k}$ represents the addition of all true negative samples, $FP=\sum_{k=1}^{n}FP_{k}$ denotes the addition of false positive, $FN=\sum_{k=1}^{n}FN_{k}$ represents the addition of False Negative.

(9) $$Accuracy=\frac{TP+TN}{TP+FP+TN+FN}$$

*Accuracy* shows the performance of the model by calculating the sum of true positive and true negative samples and then dividing it by the sum of all samples i.e., *TP*, *FP*, *TN*, and *FN* as given in Equation (9).

(10) $$Precision_{k}=\frac{TP_{k}}{TP_{k}+FP_{k}}$$

(11) $$Precision_{t}=\frac{1}{N}\;\left(\overset{n}{\underset{k=1}{\sum }}\frac{TP_{k}}{TP_{k}+FP_{k}}\right)$$

*Precision_k_* is a ratio that measures the accurateness of the model based on a negative instance fraction while *Precision_t_* calculates the total precision, which is the average of the *Precision_k_* for each class. The precision score can be obtained by the calculation of true positive samples divided by a true positive and false positive. Equation (10) shows the precision of each class while Equation (11) represents the average precision of total classes.

(12) $$Recall_{k}=\frac{TP_{k}}{TP_{k}+FN_{k}}$$

(13) $$Recall_{t}=\frac{1}{N}\;\left(\overset{n}{\underset{k=1}{\sum }}\frac{TP_{k}}{TP_{k}+FN_{k}}\right)$$

*Recall_k_* is the percentage of positive samples that are correctly identified out of all positive samples while *Recall_t_* represents the total recalls score which can be obtained from the average of *Recall_k_* for each class. Equation (12) shows the recallof each class while Equation (13) represents the average recall score of total classes.

### 4.3. Detailed Ablation Study

**Figure 6:**
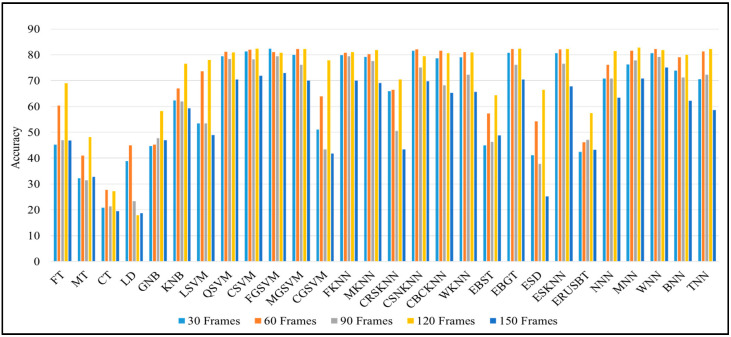
Comparison graph of different machine learning classifiers various types of sequences.

We perform extensive experiments on different machine learning models to choose the most accurate model for HAR. We evaluate the performance of different models such as FT, MT, CT, LD, GNB, KNB, LSVM, QSVM, CSVM, FGSVMMGSVM, CGSVM, FKNN, MKNN, CRSKNN, CSNKNN, CBCKNN, WKNN, EBST, EBGT, ESD, ESKNN ERUSBT, NNN, MNN, WNN, BNN, TNN, whereas the detailed performance of each model is given in Table 3 and graphical representation is demonstrated in Figure 6.

### 4.4. Deep Learning Techniques

In this section, we performed different experiments using deep learning approaches. We evaluate the performance of these models on our proposed dataset with different frames sequences. Our proposed CNN- LSTM model achieved the highest accuracy for all sequences of frames compared to other models. The experimental results of different deep learning models are shown in Table 4. For instance, the average accuracy of MLP for all types of sequences is 82.224, CNN is 84.78, LSTM is 77.53, BiLSTM is 82.624, and proposed CNNLSTM achieved 86.95 average accuracy. The proposed model achieved the highest accuracy as compared to solo deep learning-based models and traditional machine learning models as given in Table 3. The main reason behind the highest performance of the proposed model is learning spatial and temporal information from the input data while other models only extract one type of feature at a time.

**Figure 7:**
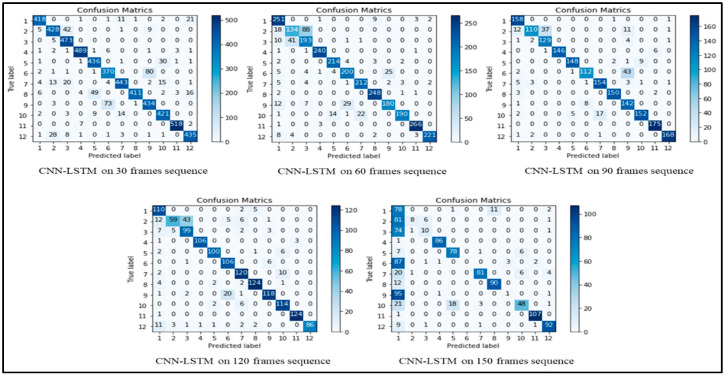
Confusion of CNN-LSTM on different frames sequences.

**Figure 8:**
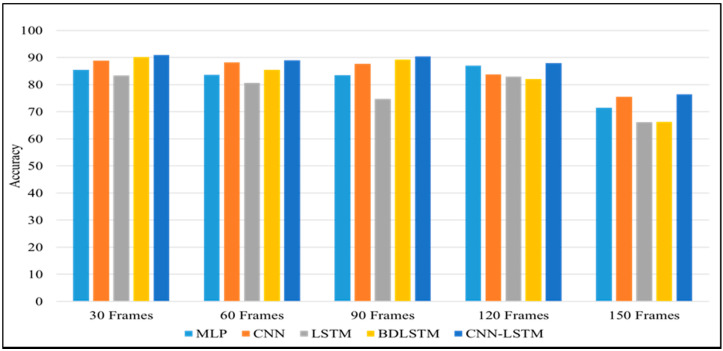
Comparison graph of the proposed model with other DL models.

From the results shown in Table 4, we can declare that the hybrid approach shows the best accuracy compared to other deep learning models. Table 5 and Table 6 show the other popular evaluation metrics i.e., precision score and recall score of our proposed techniques on different frame sequences. Figure 7 demonstrates the confusion metrics of CNN-LSTM on all five types of frames sequences and shows the TP, TN, FN and FP values of each activity. The frames sequence also depends on the accuracy of the model, if we select a very large frames sequence then it can decrease the model accuracy and performances as shown in Figure 8, for example, the performance of all models on the 150 frames sequence in Table 4 is lower than other. We used different optimizers and after investigating all optimizers we select the “Adam” Optimizer for our experiments. All the experiments are performed using the same hyperparameters such as batch size = 32, learning rate = 0.0001, and epoch = 50. These optimal parameters are selected after performing a large number of experiments on different parameters. Our model gave an excellent performance on these parameters, so we choose these parameters. The highest accuracy of 90.89% is achieved by the CNN-LSTM hybrid model on 30 frames sequence. The second highest accuracy is achieved on the 90 frames sequence. 

## 5. Conclusions

Human activity recognition through visual sensor data is a very challenging area of research from the past decades. In this paper, we propose a hybrid approach that combines CNN and LSTM to effectively recognize human activity with higher accuracy. The main purpose of using this hybrid approach in activity recognition is that human activity is actually the sequence of action that contains temporal information. CNN architecture has the advantage of extracting the discriminative features while LSTM can extracts the temporal information in time-series data. We used our own dataset which is collected from 20 participants where each participant performs 12 physical activities. This dataset contains different physical activities which can improve the individual’s health. We conducted extensive experiments on both machine learning classifier and deep learning models. We performed experiments on various machine learning classifiers such as SVM, KNN, Decision Tree, Naïve Bayes, Linear Discriminant, Ensemble classifiers (Boosted Trees, Bagged Trees, Subspace Discriminant, Subspace KNN, RUSBoosted Trees) and Neural Network (Narrow, Medium, Wide, Bi-layered, Tri-layered) on five different type of frames sequences (30 frames, 60 frames, 90 frames, 120 frames, 150 frames). We also conducted experiments on various deep learning models such as CNN, LSTM, Bidirectional LSTM, and CNN-LSTM on five different frames sequences discussed above.

In machine learning classifiers, the high accuracy is 82.4% which is achieved by three classifiers i.e., FGSVM, CSVM, and EBGT. In deep learning models, our hybrid CNN-LSTM method achieved high accuracy of 90.89% on 30 frames as compared to other deep learning approaches. The proposed hybrid model shows excellent performance on activity recognition of one-person activity, and it may not be able to perform better in the case of multiple people. In the future, we aim to increase the number of more complex physical activities and improve our model which can recognize the activity of more than one person at a time. Furthermore, we will explore advanced deep learning-based techniques such as reinforcement learning, lifelong learning, incremental and active learning for activity recognition. Additionally, we are planning to develop a huge HAR dataset that will include several daily life and physical activities.

## Figures and Tables

**Table 1:** Shows the dataset collection and activities details.

Labels	Activity Name	Participants	Time/Activity	Samples/Activity	Frame/Per Sec
1	Overhead Arm Raise	20	10 s	200	30
2	Front Arm Raise	20	10 s	200	30
3	Arm Curl	20	10 s	200	30
4	Chair Stand	20	10 s	200	30
5	Balance Walk	20	10 s	200	30
6	Side Leg Raise (Right, Left)	20	10 s	200	30
7	Shoulder	20	10 s	200	30
8	Chest	20	10 s	200	30
9	Leg Raise (Forward, Backward)	20	10 s	200	30
10	Arm Circle	20	10 s	200	30
11	Side Twist (Right, Left)	20	10 s	200	30
12	Squats	20	10 s	200	30

**Table 2:** Parameters setting of our proposed model.

Layer (Type)	Kernel Size	Filter Size	No. of Param.
1D CNN Layer 1	3	64	9664
1D CNN Layer 2	3	128	24,704
MaxPooling 1D	-	-	-
LSTM(64)	-	-	46,408
LSTM(64)	-	-	33,024
Flatten	-	-	-
Dense(12)	-	-	780
Total parameters	-	-	117,580

**Table 3:** Shows the accuracy of different machine learning classifiers on different sequences.

No.	Classifiers	Frames Sequence				
		30	60	90	120	150
1	FT	45.2	60.3	47.0	69.0	46.8
2	MT	32.3	41.0	31.4	48.1	32.7
3	CT	20.8	27.7	21.4	27.2	19.5
4	LD	38.9	45.0	23.4	17.9	18.7
5	GNB	44.7	45.2	47.7	58.3	46.9
6	KNB	62.3	67.0	62.0	76.6	59.3
7	LSVM	53.5	73.6	53.5	78.0	48.9
8	QSVM	79.4	81.2	78.4	80.9	70.5
9	CSVM	81.3	82.0	78.3	82.4	71.9
10	FGSVM	82.4	81.1	79.5	80.8	72.9
11	MGSVM	80.0	82.2	76.1	82.2	70.1
12	CGSVM	51.1	63.9	43.4	77.9	41.8
13	FKNN	79.8	80.8	79.5	81.0	70.0
14	MKNN	79.2	80.3	77.6	81.8	69.1
15	CRSKNN	65.9	66.4	50.5	70.5	43.4
16	CSNKNN	81.6	82.1	75.1	79.4	69.8
17	CBCKNN	78.6	81.6	68.2	80.6	65.3
18	WKNN	79.0	81.1	72.3	80.9	65.6
19	EBST	45.0	57.3	46.3	64.4	48.8
20	EBGT	80.8	82.3	76.2	82.4	70.4
21	ESD	41.1	54.2	37.8	66.5	25.2
22	ESKNN	80.7	82.1	76.6	82.2	67.8
23	ERUSBT	42.5	46.1	47.1	57.4	43.2
24	NNN	70.9	76.1	70.8	81.4	63.4
25	MNN	76.3	81.6	77.9	82.8	70.9
26	WNN	80.6	82.2	79.2	81.8	75.1
27	BNN	73.9	79.0	71.3	80.0	62.2
28	TNN	70.6	81.3	72.3	82.2	58.6

**Table 4:** Shows the accuracy of our hybrid approach as compared to other deep learning models.

No.	Model Name	Frames Sequence				
		30	60	90	120	150
1	MLP	85.45	83.64	83.47	87.05	71.51
2	CNN	88.82	88.22	87.65	83.74	75.47
3	LSTM	83.31	80.64	74.69	82.92	66.09
4	BiLSTM	90.15	85.39	89.30	82.02	66.26
5	CNN-LSTM	90.89	88.98	90.44	87.94	76.50

**Table 5:** The precision score of proposed techniques and other DL models on different sequences.

No.	Model Name	Frames Sequence				
		30	60	90	120	150
1	MLP	86.18	84.37	85.12	88.54	74.97
2	CNN	89.20	88.48	88.37	83.93	78.04
3	LSTM	83.94	82.51	74.95	84.04	64.01
4	BiLSTM	90.74	85.90	89.62	82.52	70.35
5	CNN-LSTM	91.11	89.31	91.13	88.82	76.13

**Table 6:** Recall Score of the proposed method and other DL models on different sequences.

No.	ModelName	Frames Sequence				
		30	60	90	120	150
1	MLP	85.39	83.43	83.58	86.86	71.92
2	CNN	88.86	88.07	87.77	83.50	75.36
3	LSTM	83.24	81.23	74.15	82.84	65.89
4	BiLSTM	90.05	85.24	89.41	82.11	67.16
5	CNN-LSTM	90.84	88.79	90.56	88.10	75.82
